# Co-vulnerabilities of inhibiting carbonic anhydrase IX in ferroptosis-mediated tumor cell death

**DOI:** 10.3389/fmolb.2023.1327310

**Published:** 2023-11-30

**Authors:** Paul C. McDonald, Shoukat Dedhar

**Affiliations:** ^1^ Department of Integrative Oncology, BC Cancer Research Institute, Vancouver, BC, Canada; ^2^ Department of Biochemistry and Molecular Biology, University of British Columbia, Vancouver, BC, Canada

**Keywords:** hypoxia, carbonic anhydrase IX, cytotoxicity, metabolism, ferroptosis, tumour microenvironment

## Abstract

The tumour-associated carbonic anhydrases (CA) IX and XII are upregulated by cancer cells to combat cellular and metabolic stress imparted by hypoxia and acidosis in solid tumours. Owing to its tumour-specific expression and function, CAIX is an attractive therapeutic target and this has driven intense efforts to develop pharmacologic agents to target its activity, including small molecule inhibitors. Many studies in multiple solid tumour models have demonstrated that targeting CAIX activity with the selective CAIX/XII inhibitor, SLC-0111, results in anti-tumour efficacy, particularly when used in combination with chemotherapy or immune checkpoint blockade, and has now advanced to the clinic. However, it has been observed that sustainability and durability of CAIX inhibition, even in combination with chemotherapy agents, is limited by the occurrence of adaptive resistance, resulting in tumour recurrence. Importantly, the data from these models demonstrates that CAIX inhibition may sensitize tumour cells to cytotoxic drugs and evidence now points to ferroptosis, an iron-dependent form of regulated cell death (RCD) that results from accumulation of toxic levels of phospholipid peroxidation as a major mechanism involved in CAIX-mediated sensitization to cancer therapy. In this mini-review, we discuss recent advances demonstrating the mechanistic role CAIX plays in sensitizing cancer cells to ferroptosis.

## 1 Introduction

Hypoxia is an important feature of the tumour microenvironment (TME) of solid cancers and its presence is associated with poor patient prognosis and resistance to anti-cancer therapies (Nakazawa et al., 2016; Gillies et al., 2018). Hypoxia is also known to provide an environmental niche for cancer stem cells and to promote invasion and metastasis (Jain, 2014). Intratumoural hypoxia promotes hypoxia-inducible factor 1 alpha (HIF-1α)-mediated metabolic reprogramming by tumour cells, resulting in a shift toward increased glycolysis and altered oxidative phosphorylation in a bid to meet energy and biosynthetic demands in a low oxygen environment (Xie and Simon, 2017). These processes lead to the accumulation of acidic metabolites by tumour cells, including lactate, protons (H^+^) and carbon dioxide (CO_2_). The aforementioned metabolites contribute to the disruption of intracellular pH (pHi) homeostasis and negatively impact cell viability (Parks et al., 2013; Corbet and Feron, 2017). Cancer cells must, therefore, actively adapt to these challenging environmental conditions in order to survive.

To effectively combat hypoxic and acidic cellular stress, cancer cells activate a network of enzymes and transporters that function to maintain pHi homeostasis (Parks et al., 2013), including the tumour-associated carbonic anhydrases (CA) IX (CAIX) and CAXII (Neri and Supuran, 2011; Corbet and Feron, 2017). CAIX is a cell surface, HIF-1α inducible metalloenzyme that catalyzes the reversible hydration of CO_2_ to bicarbonate (HCO_3_^−^) and H^+^ (Neri and Supuran, 2011). In hypoxic tumours, CAIX activity contributes to the maintenance of a pHi favorable for cancer cell survival and growth, and simultaneously facilitates acidification of the TME, thereby promoting tumour cell invasion and metastasis, as well as immunosuppression and therapeutic resistance (McDonald et al., 2012; Boyd et al., 2017; Chafe et al., 2019; Pastorekova and Gillies, 2019). Inhibition of CAIX expression disrupts pH regulation, reduces cancer stem cells, inhibits epithelial mesenchymal transition (EMT) and ultimately diminishes tumour growth and metastasis (Chiche et al., 2009; Lou et al., 2011; Lock et al., 2013; McDonald et al., 2019).

CAIX is robustly expressed in across a spectrum of hypoxic solid tumours and correlates both with poor prognosis (Chia et al., 2001; Loncaster et al., 2001; Klatte et al., 2009; Korkeila et al., 2009; Ilie et al., 2010) and with reduced therapeutic response (Koukourakis et al., 2001; Generali et al., 2006; Tan et al., 2009; McIntyre et al., 2012). In contrast, the expression of CAIX in normal human normal tissue is low and is confined to gastric and gall bladder epithelia (Supuran et al., 2018; Pastorekova and Gillies, 2019). These attributes serve to make CAIX an attractive therapeutic target, a position that, in turn, has driven the development of CAIX/CAXII small molecule inhibitors.

The 4-[(4-fluorophenyl) carbamoyl] amino-benzene sulfonamide, designated SLC-0111 (also known as U-104), is a selective small molecule inhibitor of CAIX (Pacchiano et al., 2011). Several preclinical studies have now demonstrated that targeting CAIX activity with SLC-0111 results in anti-tumour efficacy in multiple solid tumour models, including including triple negative breast cancer (Lou et al., 2011; Lock et al., 2013; Chafe et al., 2015; Bozdag et al., 2018; Hedlund et al., 2019), pancreatic cancer (McDonald et al., 2019) and melanoma (Chafe et al., 2019). Furthermore, a growing number of studies support the use of CAIX inhibitors as effective anti-cancer agents in combination with chemotherapy or immune checkpoint blockade. For example, treatment of mutant KRAS-driven PDAC tumours with the combination of gemcitabine and SLC-0111 results in acidosis and cell death, and prolongs survival by tumour-bearing mice (McDonald et al., 2019). Similarly, inhibition of CAIX activity with SLC-0111 potentiates the impact of temozolomide treatment in preclinical models of glioblastoma (Boyd et al., 2017), and combining CAIX inhibition with immune checkpoint blockade enhances anti-tumour efficacy an *in vivo* model of melanoma (Chafe et al., 2019). Mechanistically, inhibition of CAIX enhances chemo- and immunotherapeutic responses in these tumours through the regulation of pH and acidosis (Boyd et al., 2017; Chafe et al., 2019; McDonald et al., 2019). Evaluation of SLC-0111 in a Phase 1 clinical trial (NCT02215850) of patients with advanced cancer demonstrated good safety and pharmacokinetic profiles, and defined a maximum tolerated dose for Phase 2 trials (McDonald et al., 2020). SLC-0111 is currently being evaluated in combination with gemcitabine in pancreatic cancer patients with CAIX positive tumours (NCT03450018).

Furthermore, *in silico* and computational approaches may offer complementary strategies for both designing effective CAIX inhibitors and for discerning potentially actionable therapeutic combinations. For example, molecular docking and molecular dynamic simulation analyses have been performed alongside conventional fluorescence binding studies to delineate novel classes of CAIX inhibitors with efficient binding parameters (Kumari et al., 2016). To evaluate co-targeting of CAIX and other pathways relevant to cancer progression, a hybrid computational model that accounts for both tumour-immune interactions and tumour metabolism-mediated acidosis within the TME was recently developed (Grajek et al., 2023). When used to evaluate the role of CAIX expression on the efficacy of immune checkpoint inhibitors (ICI), the model showed that CAIX expression inhibits the immune response and that suppressing CAIX expression improves response to immune checkpoint blockade (Grajek et al., 2023). Computational modeling has also demonstrated that combination therapy using SLC-111 and ICI moves the incomplete response to ICI to tumour eradication (Grajek and Poleszczuk, 2023). These studies highlight the ability of *in silico* studies to recapitulate experimental findings and suggest that they may offer an additional technological link between pre-clinical studies and clinical applications.

It is clear that extensive pre-clinical and translational work, using genetic, pharmacological and *in silico* approaches has established CAIX inhibition as a promising cancer therapeutic target, especially for “difficult to treat” solid tumours. However, it has been observed that sustainability and durability of CAIX inhibition, even in combination with chemotherapy agents, is limited by the occurrence of adaptive resistance, resulting in tumour recurrence (Lou et al., 2011; Lock et al., 2013; Boyd et al., 2017; Chafe et al., 2019; McDonald et al., 2019). Importantly, the data from these models demonstrates that CAIX inhibition may sensitize tumour cells to cytotoxic drugs and evidence now points to ferroptosis, an iron-dependent form of regulated cell death (RCD) that results from accumulation of toxic levels of phospholipid peroxidation (Stockwell, 2022), as a major mechanism involved in CAIX-mediated sensitization to cancer therapy. In this mini-review, we discuss recent advances demonstrating the mechanistic role CAIX plays in sensitizing cancer cells to ferroptosis.

## 2 Co-vulnerability of iron-sulfur cluster—xCT and carbonic anhydrase IX

The acquisition of therapeutic resistance is a major impediment to durable treatment response in patients with cancer (Tyner et al., 2022). One promising therapeutic approach for overcoming treatment resistance is the identification of synthetic lethal interactions (O'Neil et al., 2017). Synthetic lethality refers to the continued survival of cells in response to a single genetic hit, whereas the co-occurrence of multiple genetic events results in cell death (O'Neil et al., 2017). Thus, identification of synthetic lethal interactions reveal co-vulnerabilities in cancer cells that may be targeted pharmacologically to generate novel therapeutic approaches.

**FIGURE 1:**
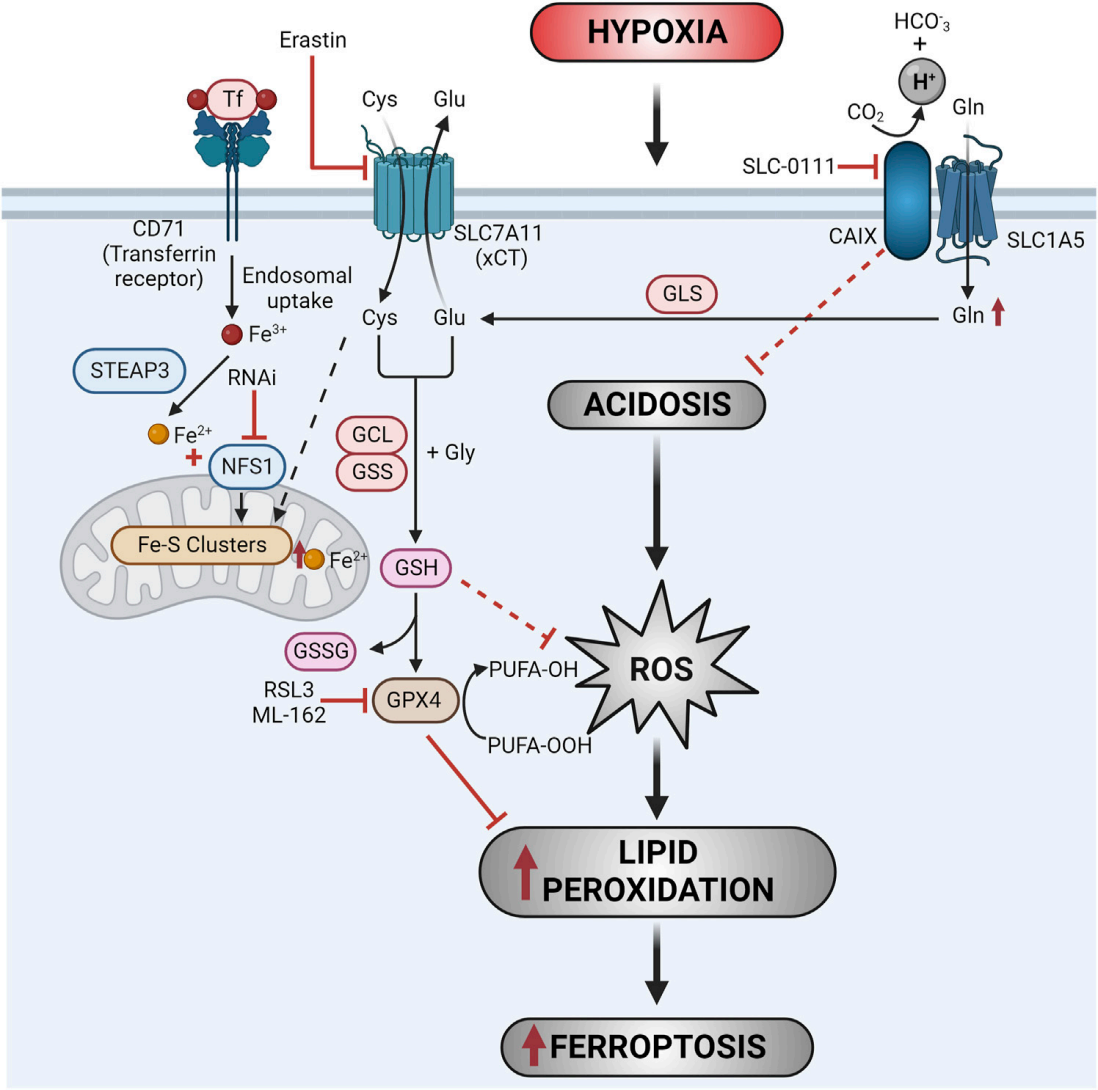
CAIX regulates tumour cell vulnerability to ferroptosis. Pharmacologic inhibition of CAIX activity, in combination with inhibition of NFS1 expression, results in intracellular acidosis and impaired iron-sulfur clustering. These events lead to increased ROS and increased labile iron which drives lipid peroxidation and increases ferroptosis. Targeting CAIX activity in combination with erastin-mediated inhibition of xCT provides an effective approach to exploiting the synthetic lethal interaction between CAIX and NFS1 using pharmacologic agents. Additionally, inhibition of CAIX increases glutamine uptake and glutathione (GSH) synthesis as a compensatory reaction to cellular stress. Co-targeting of CAIX and key metabolic nodes in glutamine metabolism and glutathione production enhances tumour cell cytotoxicity through ferroptosis. Combining CAIX/XII inhibitors with ferroptosis inducers targeting GPX4, such as RSL3 and ML-162, exploit this metabolic covulnerability to increase ferroptotic cell death.

Recently, an unbiased, genome-wide synthetic lethal CRISPR screen was performed in breast cancer cells in hypoxia to establish potential vulnerabilities that, together with inhibition of *CA9* expression, would enhance cell death and limit therapeutic resistance, offering an avenue toward suppressing tumor resistance and recurrence (Chafe et al., 2021). These analyses uncovered genes associated with redox homeostasis as co-vulnerabilities with CA9, in particular the cysteine desulfurase, NFS1, which functions to catalyze the initial step in the biogenesis of iron-sulfur clusters (Figure 1) (Chafe et al., 2021). These iron-sulfur clusters are essential cofactors for mitochondrial transport chain proteins. NFS1, which has been shown to protect cells from ferroptosis and is required for growth of metastatic breast tumours in the lung (Alvarez et al., 2017), removes a thiol group from cysteine to generate alanine and transfers the sulfur to an ISCU scaffold protein (Rouault and Maio, 2017; Maio and Rouault, 2020).

Combinatorial genetic depletion of CA9 and NFS1 expression, or pharmacologic inhibition of CAIX/XII in combination with NFS1 depletion, results in increased cellular iron pools, increased lipid peroxidation and increased ferroptosis of tumour cells (Figure 1) (Chafe et al., 2021). Furthermore, co-targeting of NFS1 and CAIX activity *in vivo* results in enhanced tumour growth control compared to targeting each protein individually (Chafe et al., 2021).

Recognizing the challenges associated with specifically targeting NFS1 for clinical application, further investigations have focused on targeting the NFS1 axis upstream through inhibition of the cell surface cysteine glutamate transporter, xCT, to limit the availability of cellular cysteine, a substrate of NFS1, in combination with inhibition of CAIX (Figure 1). Targeting CAIX activity in combination with erastin-mediated inhibition of xCT enhanced ferroptosis in cancer cells in a pH-dependent manner, providing an effective approach to exploiting the synthetic lethal interaction between CAIX and NFS1 using pharmacologic agents (Chafe et al., 2021). These studies suggest that inhibitors targeting CAIX may provide an effective strategy to enhance the activity of ferroptosis inducing compounds in cancer therapy.

Further studies have strengthened the link between CAIX and ferroptosis. The *CA9* gene was among four “hub” ferroptosis-related genes identified using machine learning and bioinformatics approaches to establish key genes and molecular interactions associated with ferroptosis in colorectal cancer (Xue et al., 2023). Interestingly, expression of CA9 was positively correlated with expression of transferrin receptor 2 (TFR2) in colorectal cancer, suggesting an association between CA9 and iron transport, which is a key process associated with ferroptosis (Xue et al., 2023). Hypoxia-mediated upregulation of *CA9* expression and increased catalytic iron (Fe^2+^) has also been observed in malignant mesothelioma (Li et al., 2019). Inhibition of CAIX activity using SLC-0111 decreased viability of malignant mesothelioma cells and induced a gene expression pattern similar to that seen with erastin-induced ferroptosis (Li et al., 2019). Inhibition of CA9 in these cells increased mitochondrial and lysosomal catalytic iron, mitochondrial ROS and lipid peroxidation. The observed cytotoxicity was significantly inhibited by Z-VAD-FMK, deferoxamine, and ferrostatin-1, suggesting that reduced cell viability occurred through a combination of apoptosis and ferroptosis (Li et al., 2019).

Evidence also suggests that combinatorial targeting of CA9 with chemotherapy in chemoresistant tumours may re-sensitize tumour cells to anti-cancer therapy, in part by enhancing ferroptosis. In gastric cancer cells, induction of chemoresistance using standard of care multi-agent perioperative chemotherapy (e.g., FLOT—Leucovorin, 5-Fluorouracil, Docetaxel, Oxaliplatin or FOLFOX—Leucovorin, 5-Fluorouracil, Oxaliplatin) resulted in increased CAIX expression, while combining these chemotherapies with SLC-0111 improved therapeutic efficacy in the drug resistant cells (Andreucci et al., 2023). While this study did not explore the relationship with ferroptosis, studies in gefitinib-resistant lung cancer, in which CAIX is upregulated, have elucidated a role of CAIX in regulating vulnerability to ferroptosis (Zhang et al., 2023). CAIX was found to provide resistance to ferroptosis-inducing drugs such as erastin through inhibition of transferrin endocytosis and stabilization of ferritin, while treatment of gefitinib-resistant lung cancer cells with CAIX inhibitor SLC-0111 (U-104) in combination with cisplatin enhanced ferroptosis *in vivo* (Zhang et al., 2023).

CAIX may also play a role in sensitizing cells to ferroptosis induced by radiation therapy. Escalating doses of ionizing radiation were observed to induce the expression of ferroptosis markers and lipid peroxidation by glioma cells (Huang et al., 2023). In this context, knockdown of *CA9* expression by glioma cells in hypoxia, an environment known to increase radioresistance of glioma cells, altered the expression of proteins involved in iron regulation and enhanced ferroptosis induced by radiation (Huang et al., 2023). Such results suggest that inhibition of CA9 may sensitize radioresistant glioma cells to ferroptosis in hypoxia.

## 3 Co-vulnerability of carbonic anhydrase IX and glutamine dependency

Metabolic plasticity by cancer cells enables the initiation of compensatory mechanisms to promote adaptation and resistance in response to therapeutic challenge (Shen et al., 2020). The contribution of CAIX to the regulation of pH and redox homeostasis by tumour cells in hypoxia positions it at an intersection with metabolic reprogramming and suggests that targeting CAIX, in combination with key metabolic nodes involved in the generation of anti-oxidants, may disrupt redox balance and sensitize cancer cells to ferroptosis.

Using triple negative breast cancer cells as a model, a recent large-scale, unbiased proteomic screen carried out to identify proteins that interact with CAIX in hypoxic cancer cells revealed that CAIX associates with the glutamine transporter, SLC1A5 (Figure 1) (Swayampakula et al., 2017). Further investigation confirmed that CAIX associates and co-localizes with SLC1A5 in cancer cells, and functions to maintain redox homeostasis through the GSH/GPX4 axis (Figure 1) (Venkateswaran et al., 2023). Inhibition of hypoxia-induced CAIX was found to increase glutamine uptake and glutathione (GSH) synthesis across a spectrum of cancer types. Importantly, co-targeting of CAIX and either Gln transport or Gln metabolism, using inhibitors of SLC1A5 or GLS and GCLC, respectively, enhanced tumour cell cytotoxicity through ferroptosis (Venkateswaran et al., 2023). Similarly, combined inhibition of CAIX and GPX4 using the ferroptosis inducer RSL3 synergistically enhanced ferroptosis and co-targeting of CAIX activity and GSH synthesis *in vivo* decreased tumour growth and increased survival through a ferrroptosis-mediated mechanism (Venkateswaran et al., 2023).

## 4 Conclusion

In conclusion, it is becoming increasingly clear that CAIX contributes functionally to the regulation of ferroptosis by cancer cells in hypoxia. While the use of CAIX inhibitors as single agents has met with some success, suppressing CAIX function in the context of large-scale unbiased genomic and proteomic approaches has revealed metabolic co-vulnerabilities that, when targeted in combination with CAIX, synergistically enhance ferroptosis of cancer cells. In the future, co-targeting CAIX/XII activity in combination with ferroptosis inducers, such as inhibitors of GPX4, have the potential to achieve substantial in-roads in treating hypoxic tumours, especially those exhibiting chemo- and radio-resistance.
